# Long Noncoding RNA *ANRIL*: Lnc-ing Genetic Variation at the Chromosome 9p21 Locus to Molecular Mechanisms of Atherosclerosis

**DOI:** 10.3389/fcvm.2018.00145

**Published:** 2018-11-06

**Authors:** Lesca M. Holdt, Daniel Teupser

**Affiliations:** Institute of Laboratory Medicine, University Hospital, LMU Munich, Munich, Germany

**Keywords:** lncRNA (long non-coding RNA), circRNA, GWAS (genome-wide association study), eQTL analysis, transcription, splicing, tumor suppressor proteins, cardiovascular diseases

## Abstract

Ever since the first genome-wide association studies (GWAS) on coronary artery disease (CAD), the Chr9p21 risk locus has emerged as a top signal in GWAS of atherosclerotic cardiovascular disease, including stroke and peripheral artery disease. The CAD risk SNPs on Chr9p21 lie within a stretch of 58 kilobases of non-protein-coding DNA, containing the gene body of the long noncoding RNA (lncRNA) *antisense non coding RNA in the INK4 locus* (*ANRIL*). How risk is affected by the Chr9p21 locus in molecular detail is a matter of ongoing research. Here we will review recent advances in the understanding that *ANRIL* serves as a key risk effector molecule of atherogenesis at the locus. One focus of this review is the shift in understanding that genetic variation at Chr9p21 not only affects the abundance of *ANRIL*, and in some cases expression of the adjacent *CDKN2A/B* tumor suppressors, but also impacts *ANRIL* splicing, such that 3′-5′-linked circular noncoding *ANRIL* RNA species are produced. We describe how the balance of linear and circular *ANRIL* RNA, determined by the Chr9p21 genotype, regulates molecular pathways and cellular functions involved in atherogenesis. We end with an outlook on how manipulating circular *ANRIL* abundance may be exploited for therapeutic purposes.

## Introduction

**Figure 1:**
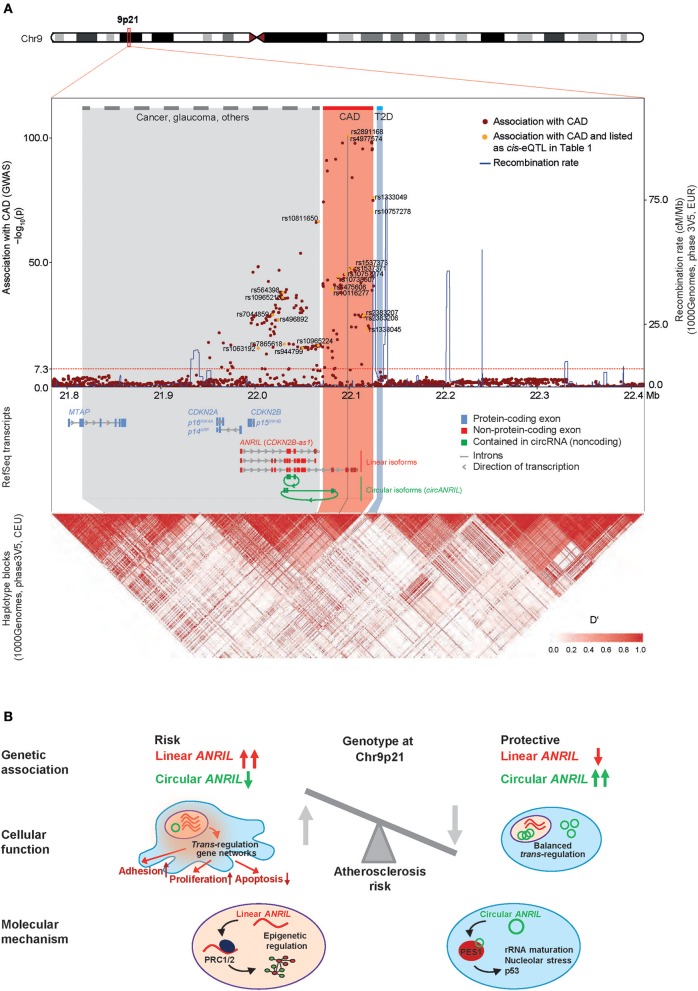
ANRIL and the CAD risk locus at Chr9p21. **(A)** Genomic mapping of SNPs identified in a region ± 300 kb around the top-ranking sentinel SNP rs4977574 based on data of the most recent large CAD GWAS (17). Chromosome ideogram and zoom-in onto RefSeq transcripts for *ANRIL, CDKN2A, CDKN2B*, and *MTAP* (**top**), regional association plot of CAD risk alleles and graph of recombination rate in the locus (**middle**), scaled linkage disequilibrium heatmap (D′) as derived from the 1000Genomes Project dataset (Phase3V5, CEU) (**bottom**). The threshold for significance of GWAS hits is indicated as horizontal dotted line (*p* < 5E-8). Dots for SNPs described in Table 1 are marked in yellow. The suspected core CAD risk region, corresponding to the distal region of *ANRIL*, has been defined experimentally through multiple CAD GWAS and is highlighted in red. The physical genomic map and the haplotype map are connected by oblique lines. Note that not all RNA transcripts and isoforms are depicted, and that type 2 diabetes (T2D, highlighted blue) and cancer risk regions (highlighted gray) are shown in simplified forms. **(B)** Model how the genotype at Chr9p21 controls the balance of linear and circular *ANRIL* RNA expression and potential molecular mechanisms of the different *ANRIL* isoforms. Linear *ANRIL* upregulation regulates gene expression *in trans* and pro-adhesive, pro-proliferative, anti-apoptotic cell functions. High levels of *circANRIL* inhibit over-proliferation of vascular cells by controlling rRNA maturation through impairing PES1 function in the PeBoW complex.

Since publication of the first genome-wide association studies (GWAS) of coronary artery disease (CAD) in 2007, Chr9p21 has emerged as the most significant risk locus associated with this frequent disease (1–4). The region contains a number of strongly interlinked SNPs within a stretch of 58 kilobases (kb) of non-protein-coding DNA. Later, the same haplotype block has been associated with other endpoints of atherosclerosis, such as stroke (5–11), peripheral artery disease (12–14), and also with different types of aneurysms (2, 8, 15, 16). Due to the availability of large study cohorts and the better resolution of genetic recombination in this region, it has now become clear that associations with other phenotypes at Chr9p21 fall in distinct haplotype blocks not overlapping with the CAD block (Figure 1A). Closely nearby, and proximal to the CAD locus, GWAS found associations with cancer, such as melanoma, glioma, basal cell carcinoma, and acute lymphoblastic leukemia [see (40) for review], and also with glaucoma, and diverse proliferative or inflammatory diseases, such as endometriosis of the reproductive tract (41), periodontitis (42), and platelet reactivity (43). The region located distally to the CAD region contains a distinct haplotype block associated with type 2 diabetes (44, 45).

In the last 10 years, GWAS have been successfully used to increase the number of genetic loci implicated in CAD risk inheritance. The number of CAD risk loci in the genome rose from 56 by 2013 (24, 46–52) to 80 by 2015 (53–56), to 243 by 2017 (17). Concerning the Chr9p21 locus in these studies, the association rose steadily from *p* = 5.40 × 10^−23^ (rs4977575) (57), over *p* = 4.68^−101^ (rs4977574) (17) to *p* = 8.8 × 10^−223^ (rs4977574) (58). In populations of European descent, the allele frequency is very high (0.48), leading to the situation that approximately one-fourth of people are homozygous for the CAD risk alleles. CAD risk SNPs on Chr9p21 have recurrently been shown to have one of the top-ranking effect sizes [allele-specific odds ratio (OR) for CAD > 1.3] (3, 24). Despite the extent of effects, the Chr9p21 risk is independent of classically known CAD risk determinants, such as dyslipidemia, diabetes mellitus, age, and sex.

The Chr9p21 region contains at least 5 genes, which are, in part, tightly clustered and overlapping. These include the 3.8 kb long *ANRIL* non-coding RNA, and the tumor suppressors *cyclin dependent kinase inhibitor CDKN2A/p16*^*INK*4*A*^, *CDKN2A/p14*^*ARF*^, *CDKN2B/p15*^*INK*4*B*^, and *methylthioadenosine phosphorylase* (*MTAP*)*. ANRIL* overlaps in antisense the full length of the *p15* gene body, while sharing a bidirectional promoter with *CDKN2A*. Hence, it was also termed *CDKN2B antisense RNA* (*CDKN2B-AS1*). Only recently, the picture got even more complex: Advances in high-throughput sequencing and adaptions in bioinformatics mapping of RNA reads to reference genomes have revealed that thousands of genes in our genome produce not only mature linear RNA but also 3′-5′ covalently linked circular RNAs (circRNAs) (59). So far, two studies have shown that a number of circular *ANRIL* (*circANRIL*) isoforms exist, comprised of different exons, whereby a downstream exon is fused to an upstream exon by the enzymatic activity of the spliceosome in a reaction termed “backsplicing” [see (60, 61) for review]. Circularizing exons in *ANRIL* stemmed mostly from middle parts of the lncRNA (Figure 1A), which are in part also shared by the linear *ANRIL* isoforms. *CircANRIL* was found not only in many different cell lines, but also in many primary cell types, including vascular smooth muscle cells (VSMCs) and macrophages, as well as in heart and vascular tissue (22, 36).

A major focus in exploring how risk is effected by Chr9p21 has been on whether genetic variation affected expression of genes at the locus *in cis* (Figure 1A) or whether it elicited gene expression changes *in trans*. Top CAD-associated SNPs lie within the distal parts of long linear *ANRIL* isoforms (Figure 1A) and several studies have shown that they co-localize with sequences marked by chromatin modifications, RNA polymerase II transcription patterns and DNA motifs characteristic of *bona-fide* transcriptional enhancers (19, 35, 62–65). Using expression quantitative trait locus (eQTL) analyses in patient samples, several groups have by now investigated if the risk alleles at the locus were associated with the expression of specific target genes *in cis* (cis-eQTLs). Whereas studies investigating *ANRIL* expression have mostly used quantitative PCRs (qPCRs) targeting different exons from the lncRNA, expression of *p14, p15, p16*, or *MTAP* has either been investigated using genome-wide expression arrays or isoform-specific qPCRs. Here, we focus on studies investigating eQTLs in atherosclerosis cohorts but do not cover studies related to other phenotypes, such as cancer, which are reviewed elsewhere (66).

## *Cis*-eQTLs at Chr9p21

*ANRIL* expression at Chr9p21 is complex and at least 20 linear isoforms as well as multiple circular isoforms have been reported [www.ensembl.org, (22, 36, 39)]. In principal, linear and circular isoforms can be distinguished by the fact that the latter derive from a backsplice event, where splicing of a downstream exon (e.g., exon 7) to an upstream exon (e.g., to exon 5) can be detected. Backsplicing of ex7-5 was the most common event observed in our own study in peripheral blood monocytes (36). Concordantly, Burd and colleagues have reported dominant backsplice isoforms spanning ex14-4 in peripheral blood T lymphocytes (22). In both studies, exon 1 and exons 17-20 were not contained in circularized *ANRIL* (Table 1). Thus, for classification reasons, results from studies targeting these exons will be referred to as proximal linear isoforms (containing the first *ANRIL* exons) and long linear isoforms (containing the distal exons 17-20) (Table 1). Since both linear and circular *ANRIL* may contain exons from the middle portion of the lncRNA (e.g., exons 4-16), a clear distinction as to whether linear or circular isoforms were investigated cannot be made in cases where these exons were targeted by qPCRs which were non-specific for backsplice junctions (Table 1).

**Table 1:** Chr9p21 cis-eQTLs in patients.

					***ANRIL*** **17 linear and dozens of circular isoforms**				***CDKN2B***	***CDKN2A***		***MTAP***

					Proximal exons1-5^*^	**Middle exons 4-16**		**Distal exons 17-20**				
**References**	**CAD SNPs**	**Cells or tissues**	**Sample size**	**Assay**	Linear^*^	**Linear or circular**	Circular^**^	**Linear**	***p15***	***p16**^*INK*4*A*^*	***p14**^*ARF*^*	
(18)	rs10757278	Peripheral blood T cells (healthy)	170	qPCR	n.d.	ex4-5	n.d.	n.d.				
(19)	rs10757272, rs4977574, rs2891168, rs133048, rs1333049	Whole blood (healthy)	120	qPCR	ex1-5	ex13	n.d.	n.d.				n.d.
						ex15-16						
		Whole blood (CAD)	42	Microarray, qPCR	n.d.	ex13				n.d.		
(20)	rs2891168, rs2383207, rs2383206, rs1333049, rs1333045, rs10757278, rs10757274, rs10116277	Lymphoblastoid cell lines from HapMap	233	Microarray	ex1–3	ex6,7,14	n.d.	ex17–19				
		Vascular tissue	294		ex1–3	ex6,7,14	n.d.	ex17–19				
(13)	SNPs rs10757274, rs2383206, rs2383207, rs10757278; rs10738605	PBMCs (healthy, CAD)	1098	qPCR	ex1-5	ex4-5	n.d.	ex18-19				
		Whole blood (healthy, CAD)	769		ex1-5	ex4-5	n.d.	ex18-19				
		Vascular tissues	41		ex1-5	ex4-5	n.d.	ex18-19				
(21)	rs10757274, rs10757278, rs1333049	Whole blood (healthy)	487	qPCR	ex1-2	n.d.	n.d.	n.d.				n.d.
	rs2383206				ex1-2							
	rs3217992				ex1-2							
	rs7044859, rs496892, rs564398 and others				ex1-2							
(22)	rs10757278	Peripheral blood T cells (healthy)	106	qPCR	ex1-2	n.d.	ex14-5 circular	ex18-19	n.d.	n.d.	n.d.	n.d.
(23)	rs1075727	PBMCs (healthy/subclinical CAD)	1490	Microarray	n.d.	n.d.	n.d.	n.d.	n.d.			n.d.
(24)	rs4977574	Omental adipose tissue (healthy, CAD)	2430	Microarray	n.d.	n.d.	n.d.	n.d.		n.d.	n.d.	n.d.
(25)	rs10757274, rs2383206, rs2383207	Vascular tissue (CAD)	57	qPCR	n.d.	n.d.	n.d.	n.d.				
(26)	rs4977574	aortic SMCs (healthy)	79	qPCR	n.d.	n.d.	n.d.	n.d.				
(27)	rs1333049	Heart (healthy/subclinical)	108	Microarray, qPCR	ex1-2	n.d.	n.d.	n.d.				
		Combined analysis of myocardium, vascular tissues (healthy, CAD)	406	Microarray	ex1-2	n.d.	n.d.	n.d.				
(28)	rs10757274	whole blood (healthy, CAD)	205	qPCR	ex1-5	n.d.	n.d.	ex17-18				n.d.
					ex4-5							
(29)	rs496892, rs564398, rs6475606, rs1063192, rs10811650, rs10738607 (prox. *ANRIL*); rs10965235, rs3731217 (dist. *ANRIL*); rs2383208 (*CDKN2B*)	Whole blood (healthy, CAD)	57	qPCR	ex1-2	n.d.	n.d.	ex17-18		n.d.	n.d.	n.d.
	rs564398, rs10965224 (short *ANRIL*); rs2811712, rs7855162 (dist. *ANRIL*); rs10965235 (*CDKN2A*)				ex1-2			ex17-18	n.d.		n.d.	n.d.
	rs1333049				ex1-2			ex17-18	n.d.	n.d.	n.d.	n.d.
(30)	rs7044859, rs496892 rs7865618, rs1333049	PBMCs differentiated to macrophages (healthy, CAD)	68	microarray, qPCR	n.d.	n.d.	n.d.	n.d.				
(31)	rs1333049	Primary VSMCs (arterial from umbilical cords, healthy)	69	qPCR, Western, histochemistry	n.d.	ex14-15	n.d.	n.d.				n.d.
(32)	rs944799 (ex12-13 *ANRIL*)	PBMCs (healthy, CAD)	281	qPCR	n.d.	ex12-13	n.d.	n.d.			n.d.	n.d.
	rs944799, rs598664, rs1002878 (*ANRIL*); rs598664 (*CDKN2B*)				n.d.	ex13-14	n.d.	n.d.			n.d.	n.d.
	rs10965228 (*ANRIL*)	Platelet-rich blood plasma (without PBMCs)			n.d.	ex12-13	n.d.	n.d.			n.d.	n.d.
						ex13-14						
(33)	rs10757274, rs2383206, rs2383207, rs10757278	PBMCs (healthy, CAD)	1933	microarray, qPCR	ex 1-5	ex7-13	n.d.	ex18-19				
		Whole blood (healthy, CAD)	960		ex 1-5	ex7b, ex7-13, ex1-5, ex10-13b	n.d.	ex18-19				
		Vascular tissues	193		ex 1-5	ex7b, ex7-13, ex1-5, ex10-13b	n.d.	ex18-19				
(34)	rs10757278, rs10757274, rs2383206, rs1333049, rs4977574	Whole blood; plasma/healthy, CAD)	200	qPCR (blood), ELISA (plasma)	ex1-5	n.d.	n.d.	n.d.	n.d.		n.d.	n.d.
(36)	rs10757274, rs2383206, rs2383207, rs10757278	PBMCs (healthy, CAD)	1933	Microarray, qPCR	n.d.	n.d.	ex7-5 circular	n.d.				
		Whole blood (healthy, CAD)	1933		n.d.	n.d.	ex7-5 circular	n.d.				
		Vascular tissues	218		n.d.	n.d.	ex7-5 circular	n.d.				
(37)	rs1537371, rs1333040	Vascular tissues, fat, muscle, blood (CAD)	600	RNAseq	n.d.				^#^	n.d.	n.d.	n.d.
(18)	rs10965215, rs10738605	PBMCs (healthy, CAD)	66	qPCR	n.d.	ex7	n.d.	n.d.	n.d.	n.d.	n.d.	n.d.
(17)	rs1537371	aorta (CAD)	600	RNAseq	n.d.	n.d.	n.d.	n.d.		n.d.	n.d.	n.d.
		Total number of relevant studies			10	8	2	6	19	18		10
		% of studies showing upregulation			70	37	0	50	11	6		0
		% of studies showing downregulation			30	50	100	17	47	17		0
		% of studies showing unchanged expression			40	63	0	67	63	78		100

*CAD-relevant SNPs encoded in Chr9p21 and defined as cis-eQTLs for regulation of ANRIL, p15, p16, p14, and/or MTAP through gene expression profiling with microarrays or targeted qPCR analysis*.

*Upregulation of target gene expression (green), downregulation (red), no effect on RNA abundance (gray), effect not determined (n.d.). Effects on different isoforms of ANRIL (linear or circular) is specified when exon-specific qPCR analysis was performed targeting proximal, middle or distal ANRIL exons (ex). In total 23 studies are reviewed here. References: (13, 17–38). Note that the percentages (%) of studies showing up- and down-regulation of ANRIL isoforms do not necessarily add up to 100%, because different ANRIL exons were quantified in different studies, not all classes of ANRIL transcripts were analyzed in each study, and because one study can report on both up- and downregulation of different isoforms belonging to the same class of ANRIL transcripts (proximal/middle/distal)*.

* *(ANRIL linearity determined by PCR forward primer residing in exons 1, 2, or 3)*.

** (ANRIL circularity determined by PCR primers detecting backsplicing between exons 10-2, 5-intron3, 6-intron3, 6-4, 7-4, 14-4, 10-4, 12-4, 13-4, 14-4, 16-4, 6-5, 7-5, 8-5, 10-5, 14-5 16-5, 19-5, 7-6, 10-6, 14-6, 16-13^*^, 16-15) (22, 36, 39).

# *(cis-eQTL locating in enhancer element, but with unspecified direction of effect on expression)*.

As one of the first studies on Chr9p21, Jarinova et al. have shown that *ANRIL* expression was induced by the CAD risk SNP rs1333049 in peripheral blood monocytes (PBMCs). No significant effects on *CDKN2A* or on *CDKN2B* were recorded in that study (19). Over the years, comparable quantifications of these genes followed in whole blood, peripheral blood T lymphocytes, lymphoblastoid cells lines, aortic smooth muscle cells (SMCs) and in different tissue samples that are known to have a role in atherosclerosis. For example, vascular tissues such as carotid atherosclerotic plaque samples, samples from aorta, mammary artery, and from the heart ventricles have been analyzed, but also tissues like subcutaneous or omental fat have been used (Table 1). Of the 23 cis-eQTL studies conducted in the Chr9p21 CAD region to date, 16 investigated different isoforms of *ANRIL*, out of which 10 used assays targeting proximal *ANRIL* exons, 8 used assays targeting the middle region, 6 used assays targeting downstream linear *ANRIL* exons, and two investigated backsplices contained in *circANRIL* (Table 1). Complicating a clear-cut interpretation, in the different studies, different risk genotypes were used to indicate risk haplotypes. The expression of *CDKN2A* and of *CDKN2B* was investigated in 18 studies and *MTAP* in 10 studies (Table 1).

Overall, 80% of the studies investigating *ANRIL* expression found an association with the Chr9p21 genotype. Here, a trend toward higher expression of the proximal and distal exons contained in linear *ANRIL* in patients carrying the CAD-risk allele was observed (7 of 10 and 3 of 6 studies). In contrast, circular *ANRIL* was downregulated in the two published studies in patients carrying the Chr9p21 risk haplotype. No clear tendency was observed when assays targeting the middle region of *ANRIL* were used (Table 1). This is likely explained by the fact that these assays target both, linear and circular, *ANRIL* isoforms, which seem to be inversely regulated. With respect to the tumor suppressor genes contained at the Chr9p21 locus, 78 and 67% of the studies failed to find an association of *CDKN2A* and *CDKN2B* with Chr9p21, respectively. When reporting an association, specifically *CDKN2B* was down-regulated in the majority of studies (94%), yet its expression was not always anticorrelating with *ANRIL* expression (19, 21, 22, 29). *MTAP* expression was not associated with the Chr9p21 genotype in any of the published studies. Overall, the picture emerges that circular *ANRIL* and *CDKN2B* tend to be down-regulated in patients carrying the risk allele, whereas linear *ANRIL* isoforms tend to be inversely regulated (Figure 1B). It is currently unclear, why expression of *p15* or of *p14* and *p16* were in many cases positively correlated with *ANRIL* (19, 21, 22, 27, 29, 32, 34, 65). Also, *MTAP*, which was not associated with Chr9p21 (Table 1), was in some conditions anticorrelating to *ANRIL*, but not in all cases or contexts (20, 34, 67). SNPs in *ANRIL* can hypothetically affect enhancers in both directions, either by disrupting transcription factor binding sites in open chromatin (68) or by increasing enhancer activity through yet unknown primary effects (24, 65).

In summary, many studies document *cis*-eQTLs for *ANRIL* or, separately, for *CDKN2B* (35). Throughout, from the existing data, it can be concluded that these effects are cell-type specific and combinatorial. Of note, many studies have investigated only very small cohorts and those, simultaneously testing both *ANRIL* and *CDKN2B* in larger cohorts (>1000 samples) identified much stronger effects of Chr9p21 on *ANRIL* than on *CDKN2B* (13, 33, 36). This observation might be explained by the haplotype block structure of the region, where effects of CAD lead SNPs are located within *ANRIL* but bleed through due to linkage disequilibrium, resulting in more subtle concomitant effects on *CDKN2B* expression. Another possibility is that the Chr9p21 genotype impacts transcription enhancers at the locus which contact and activate gene promoters affecting CAD. The consequences of such contacts would not be expected to be captured through traditional non-allelic RNA expression analysis. In fact, when allelic expression control through 3D-enhancer looping was specifically measured in a separate study in human coronary aortic SMCs (64), physical contacts of CAD variant-containing enhancers in the locus and the promoters of *CDKN2A, CDKN2B*, and *ANRIL* were corroborated.

Taken together, these data suggest that genetic variation within the core 9p21 CAD region relates to differential expression not only of *ANRIL*, but in specific cells or conditions, also of the *CDKN2A/B* tumor suppressors encoded in the locus. While either of these factors could potentially increase cell proliferation, or lead to unscheduled senescence, or elicit out of context inflammatory signaling, as far as based on work with cells *in vitro*, no study in humans or in mouse models has been able to decisively implicate a downstream effector pathway *in vivo*.

## *Trans*-eQTLs at Chr9p21 and molecular functions of *ANRIL* in transcriptional regulation

As opposed to *cis* effects, two eQTL studies have so far detected modest and tissue-selective differential expression of dozens of genes associated with Chr9p21 genotype with genome-wide significance (19, 27). Affected genes were from a broad range of classes (*AVPR2, PEAK1, FBLN1, KALRN, DAZL, STAU2, HLA-DQA1, BTNL8, PLEKHA6, TDGF1*) in whole blood (19) and different, non-overlapping gene sets linked to tissue wounding, cell migration and inflammatory response, when analyzing heart tissue, plaques, aortas, and arteries (27).

Other, and in part, larger studies in vascular tissue (20), peripheral blood mononuclear cells (PBMC, *n* = 2280) (33) and in blood monocytes (*n* = 1490) (23) reported no significant expression association.

Though not directly comparable, another study showed that in macrophages cultured *in vitro* under stress-bearing IFNγ and LPS stimulation, the CAD risk genotype led to differential up- and downregulation of target genes outside the Chr9p21 locus and yet distinct from the previously mentioned studies (*IL1B, IL12B, CASP5, CCL8, MT1A, MT1E, MUCL1, TNIP3, VCAN, ENPP2, NDP, CD163)* (30). Also *ANRIL* knockdown in cultured cell lines (69–72) and overexpression of linear *ANRIL* affected the expression of non-overlapping gene sets in the genome *in trans* (33, 36).

How *ANRIL* exerts *trans*-regulation is not known, and despite a study that showed a physical interaction of *ANRIL* with promoters of target genes (33), this role is likely not a classical function as enhancer RNA [eRNA (73)], because it involved both up-and down-regulated genes, and was suggested to involve sequence homology (33). In the case of *ANRIL, trans*-regulation of target genes was ascribed to an ALU motif in both *ANRIL* and the target gene promoters (33). Similarly, an independent study found that *ANRIL* did not only silence its targets, but unexpectedly also upregulated target genes: For example, proinflammatory interleukins *IL6/8* were found to be co-stimulated by *ANRIL* and YY1, a transcription-regulating factor that bound to the *ANRIL RNA*, especially in the context of TNFα/NFκB signaling (70). Therefore, opposite to what could have been expected from the reported physical interaction of *ANRIL* with proteins from the repressive Polycomb group complexes (74), *ANRIL* might be an activator, at least for some *trans*-regulated genes (33, 70) (see chapter 4 for details). Whether *circANRIL*, beyond regulating rRNA maturation, is involved in primary transcriptional control, alone or via impacting linear *ANRIL*'s function, is not known (36). Nevertheless, it is interesting to note that *circANRIL* isoforms linked to CAD are produced from exons located in the middle of the *ANRIL* gene (22, 36), and as such do not include the ALU motif, which is important for gene *trans*-regulation by linear *ANRIL* and is located more distally in the gene (33). Thus, variation in *ANRIL* RNA at the molecular level (linear vs. circular) might impose a fundamental alteration in *ANRIL* effector function, while not offering any explanation *per se* on how linear *ANRIL* regulates genes, as scaffold for promoter-activating complexes, or as decoy/inhibitor of repressive chromatin-modifying complexes. Conservatively speaking, it seems possible that Chr9p21 CAD risk genotypes affects genomic expression both *in cis* and *in trans*, and linear *ANRIL* RNA may be one, but not the sole, important effector molecule for how the Chr9p21 locus transduces such effects (Figure 1B).

## Correlation of Chr9p21 genes with atherosclerosis severity in humans and mouse models

Another piece of evidence for a functional role of *ANRIL* in determining CAD risk stems from correlation analysis with disease features in patient cohorts. Aside of the genetic association, *ANRIL* levels were often increased in CAD patients, and not only in atherosclerotic plaque tissue, but also in circulating PBMCs or whole blood. Here, linear *ANRIL* levels were positively correlated with the severity of atherosclerosis (13, 29, 75) whereas *circANRIL* was anticorrelated (36) (Figure 1B). Thus, while the genotype of Chr9p21 determines the production of atherogenic (linear) over antiatherogenic *ANRIL* RNA species (circular), CAD and peripheral artery disease-dependent changes may additionally feed into *ANRIL* regulation. For *CDKN2B*, two studies reported a correlation of the expression with atherosclerosis severity (34, 76), where the direction of the correlation (downregulation in plaques) was consistent from what could be expected from the association results. But another study reported increased *p16*^*INK*4*A*^ levels to positively correlate with inflammation markers in plaques instead of anticorrelation (25). Together, results from association as well as correlation analyses have etablished *ANRIL* lncRNA as prime candidate at the Chr9p21 locus.

## Molecular function of *ANRIL* and *CDKN2A/B* in atherogenesis

*ANRIL* belongs to the group of long non-coding RNAs and as such has been suggested to act as a molecular scaffold of chromatin-modifying complexes that control gene expression through modifying histone tails. Specifically*, ANRIL* was found to physically interact with the CBX7 protein inside the PRC1 Polycomb complex, one of the major gene repression complexes in cells (74). Knockdown of members of this Polycomb group complex led to increased expression of the *CDKN2A* and *CDKN2B* tumor suppressors in the Chr9p21 locus. Also, ongoing RNA polymerase II transcription was important for the association of the Polycomb proteins with the locus, indicative of the importance of RNA for recruitment. It was concluded that *ANRIL*'s function may be, at least in part, to repress the *CDKN2A* and *CDKN2B* tumor suppressors. As a consequence increased *ANRIL* levels are thought to promote overproliferation and to be incompatible with senescence onset, a major function of *CDKN2A/B*. As described in chapter 2, other work has shown that recruitment of the Polycomb complexes may account also for how *ANRIL* regulates genes *in trans* on a genome-wide level: Overexpression of linear *ANRIL* isoforms in cultured cells was found to promote pro-atherogenic cell functions, such as proliferation and reduced apoptosis, and to trigger the differential expression of hundreds of genes, in this case without affecting *CDKN2A/B* suppressors. Results from that study therefore questioned whether *ANRIL* regulated these tumor suppressor genes *in cis* at all (36, 77).

How does circular *ANRIL*, whose abundances is reduced in CAD patients, fit into this model? Both in human peripheral blood T-lymphocytes, as well as in PBMCs, whole blood and endatherectomy plaque tissue, *circANRIL* isoforms were found to be downregulated in samples from CAD patients carrying the Chr9p21 risk allele (22, 36). In an initial model, it was suggested that the production of *circANRIL* from central *ANRIL* exons would shorten the linear *ANRIL* lncRNA and, thereby, impaired linear *ANRIL's* function in epigenetic control of target genes (22). In a second study, a more primary role was found for *circANRIL* that was, furthermore, independent of linear *ANRIL* (36). Here, *circANRIL* was found to be 10-fold more abundant than linear *ANRIL*. Mass-spectrometric analysis of proteins interacting with *circANRIL* showed that it bound to PES1 protein, a member of the evolutionarily conserved PeBoW complex. This complex is essential for proper rRNA-processing, that is the excision of RNA spacer elements from pre-ribosomal rRNA precursors. *CircANRIL* inhibited the activity of the PeBoW complex, as deduced from the accumulation of unsufficiently processed (and non-functional) 26S and 32S pre-rRNA intermediates when *circANRIL* was overexpressed (36). A deficit in rRNA maturation caused nucleolar stress and p53 activation, culminating in inhibition of cell proliferation and in an increase in apoptosis. Notably, the observed functions of *circANRIL* were inverse to that of linear *ANRIL* and, as shown by genomic knockout of linear *ANRIL* exons, independent from the presence of these lncRNA isoforms. Thus, experimental evidence from expression analysis *in vivo* and from genetic experiments both indicated that *circANRIL* was anti-atherogenic. Together, linear *ANRIL* confers overproliferation, and circular *ANRIL* protects from overproliferation, suggesting that the genotype of Chr9p21 is important to determine the balance of linear and circular *ANRIL* levels in SMCs and macrophages, and that a dominance of linear *ANRIL* in this ratio, even when small, over decades skews for CAD (36) (Figure 1B).

Whether suppressing linear *ANRIL* or boosting circularization is sufficient to protect from atherosclerotic cues *in vivo* is matter of ongoing research. The fact that *ANRIL* RNA is not conserved beyond primates complicates the functional *in vivo* analysis of the Chr9p21 locus. So far, insight on how CAD is controlled by Chr9p21 through genetic modeling in mouse mutants is fragmented. The genetic elements of Chr9p21 and their relative positioning are overall syntenically conserved in mouse chromosome 4. So far, only one study has investigated, if deletion of a 70 kb long portion of mouse Chr4 corresponding to the CAD haplotype block in humans had an effect on atherosclerosis *in vivo* (78). This region contains a multi-exon lncRNA, *AK148321*, which is, however, likely not corresponding to human *ANRIL*. Mutant mice (78) developed tumors, reminiscent of tumorigenesis associated with mutation in the Chr9p21 region. But despite some metabolic changes in the mutant mice and enhanced platelet activation, no significant change in atherosclerotic fatty lesion formation was observed (78), putting in question the validity of this mouse model for studying *ANRIL*-driven atherogenesis. On the other hand, the mutants did develop more vascular aneurysms (79), supporting that some aspects of CAD were indeed contained in the noncoding mouse sequence.

Overall, the picture is not yet fully clear. While the genetic data from mice support the importance of individual noncoding genetic elements and of some of the protein-coding tumor suppressors for regulation of atherosclerosis and other CAD entities, whether the lncRNA encoded in the locus regulates CAD mechanistically via epigenetically regulating the neighboring tumor suppressors *in cis* has not been determined. Nevertheless, mouse genetics remains an interesting research avenue to explore some aspects of Chr9p21 biology, at least relating to aneurysm, cancer, and glaucoma formation.

## Summary

Starting from a GWAS signal for CAD in a “gene desert” on Chr9p21 in 2007, research in the last decade has firmly established this region as strongest genetic factor of human atherosclerosis and has contributed to a better understanding of the underlying pathophysiology. The picture has emerged that one of the major routes how this locus controls atherosclerosis risk is through regulating the expression of the lncRNA *ANRIL in cis*, where the risk allele leads to high levels of linear *ANRIL* but decreases circular *ANRIL* expression. Linear *ANRIL* has been established as molecular scaffold guiding epigenetic protein complexes and promoting pro-atherogentic cells functions. On the contrary, circularization shifts *ANRIL's* function toward controlling ribosomal RNA processing and controlling protein translation thereby promoting athero-protection (Figure 1B). The molecular mechanisms of how the ratio of linear and circular *ANRIL* is controlled by the genotype at the locus are currently not resolved and it will be important to determine which gene regulatory elements within the *ANRIL* gene are disturbed by causal CAD risk SNPs. Experimentally exploring details of the molecular effector mechanisms for linear *ANRIL* and for circular *ANRIL* will be paramount, but this task will not be trivial because linear and circular *ANRIL* isoforms always co-exist and in part share the same sequence. Not last, more nuanced relations between Chr9p21 genotype and gene expression output can be expected to be found in the future if, for example, analyses were to take into account cell type-specific and context (stress, inflammation, senescence)-specific effects, aspect that whole tissue expression profiling is currently missing. Additionally, although it is early days, measuring the levels of *circANRIL/linear ANRIL*, might offer a prognostic value and help improve CAD risk stratification or allow to better monitor treatment response or disease recurrence.Yet, since *circANRIL* levels are reduced in plaque tissue, and since *circANRIL* has been found to be anti-atherogenic with or without co-existing linear *ANRIL*, increasing *circANRIL* abundance in patients could also be of therapeutic relevance. Expressing *circANRIL* levels in the cells of the vasculature in CAD disease models might, therefore, be a promising next step to exploit the accumulated knowledge on the Chr9p21 CAD risk locus.

## Author contributions

All authors listed have made a substantial, direct and intellectual contribution to the work, and approved it for publication.

### Conflict of interest statement

The authors declare that the research was conducted in the absence of any commercial or financial relationships that could be construed as a potential conflict of interest.
